# Fenofibrate Protects Cardiomyocytes from Hypoxia/Reperfusion- and High Glucose-Induced Detrimental Effects

**DOI:** 10.1155/2021/8895376

**Published:** 2021-01-09

**Authors:** Fabiola Cortes-Lopez, Alicia Sanchez-Mendoza, David Centurion, Luz G. Cervantes-Perez, Vicente Castrejon-Tellez, Leonardo del Valle-Mondragon, Elizabeth Soria-Castro, Victoria Ramirez, Araceli Sanchez-Lopez, Gustavo Pastelin-Hernandez, Wylly Ramses Garcia-Niño, Maria Sanchez-Aguilar, Luz Ibarra-Lara

**Affiliations:** ^1^Department of Pharmacobiology, Center for Research and Advanced Studies of the National Polytechnic Institute, Calz. de los Tenorios 235, Col Granjas Coapa, Tlalpan, 14330 Mexico City, Mexico; ^2^Department of Pharmacology, National Institute of Cardiology Ignacio Chávez, Juan Badiano No. 1, Col. Sección XVI, Tlalpan, 14080 Mexico City, Mexico; ^3^Department of Physiology, National Institute of Cardiology Ignacio Chávez, Mexico City, Mexico; ^4^Department of Cardiovascular Biomedicine, National Institute of Cardiology Ignacio Chávez, Mexico City, Mexico; ^5^Department of Experimental Surgery, National Institute of Medical Sciences and Nutrition Salvador Zubirán, Vasco de Quiroga 15, Belisario Domínguez, Col. Sección XVI, Tlalpan, 14080 Mexico City, Mexico

## Abstract

Lesions caused by high glucose (HG), hypoxia/reperfusion (H/R), and the coexistence of both conditions in cardiomyocytes are linked to an overproduction of reactive oxygen species (ROS), causing irreversible damage to macromolecules in the cardiomyocyte as well as its ultrastructure. Fenofibrate, a peroxisome proliferator-activated receptor alpha (PPAR*α*) agonist, promotes beneficial activities counteracting cardiac injury. Therefore, the objective of this work was to determine the potential protective effect of fenofibrate in cardiomyocytes exposed to HG, H/R, and HG+H/R. Cardiomyocyte cultures were divided into four main groups: (1) control (CT), (2) HG (25 mM), (3) H/R, and (4) HG+H/R. Our results indicate that cell viability decreases in cardiomyocytes undergoing HG, H/R, and both conditions, while fenofibrate improves cell viability in every case. Fenofibrate also decreases ROS production as well as nicotinamide adenine dinucleotide phosphate oxidase (NADPH) subunit expression. Regarding the antioxidant defense, superoxide dismutase (SOD Cu^2+^/Zn^2+^ and SOD Mn^2+^), catalase, and the antioxidant capacity were decreased in HG, H/R, and HG+H/R-exposed cardiomyocytes, while fenofibrate increased those parameters. The expression of nuclear factor erythroid 2-related factor 2 (Nrf2) increased significantly in treated cells, while pathologies increased the expression of its inhibitor Keap1. Oxidative stress-induced mitochondrial damage was lower in fenofibrate-exposed cardiomyocytes. Endothelial nitric oxide synthase was also favored in cardiomyocytes treated with fenofibrate. Our results suggest that fenofibrate preserves the antioxidant status and the ultrastructure in cardiomyocytes undergoing HG, H/R, and HG+H/R preventing damage to essential macromolecules involved in the proper functioning of the cardiomyocyte.

## 1. Introduction

Diabetes is becoming alarmingly prevalent, raising the cardiovascular risk to develop myocardial ischemia and chronic heart failure with poor prognosis and survival [1]. The impaired glucose metabolism and the exacerbation of ischemia-reperfusion injury in diabetic myocardium enhances the production of oxygen reactive species (ROS), giving rise to oxidative stress and leading to biochemical and structural changes in detriment of cardiac function [2, 3].

Hyperglycemia and hypoxia-induced overproduction of anion superoxide damages organelles and macromolecules important for cell survival like lipids, enzymes, proteins, and DNA [4]. A key participant in this process is the activation of various NADPH oxidase isoforms in cardiovascular cells [5, 6]. In this regard, the overproduction of ROS has been widely reported to promote the uncoupling of eNOS dimer; further increasing superoxide anion production [7]. Additionally, the presence of hypoxia-inducible factor-1 (HIF-1*α*) is increased in response to hypoxia, in order to regulate a plethora of genes that help cells to cope with oxygen restriction [8]. In order to decrease ROS production, it is necessary to activate and/or increase the antioxidant defense or to diminish oxidative stress [9]. Therefore, novel therapeutic strategies are required to protect the myocardium against the effects of ischemia-reperfusion (I/R) injury in hyperglycemic conditions.

A pharmacological target to counteract oxidative stress-induced damage may be peroxisome proliferator-activated receptors (PPARs) which are nuclear proteins acting as transcription factors [10, 11]. Besides a role in lipid metabolism PPARs play a number of pleiotropic effects, that include a critical role in myocardial health, and influence the production of cytokines and growth factor release, leading to anti-inflammatory and anti -proliferative effects [12]. The three main types of PPARs are encoded by separate genes and their products have been identified as PPAR alpha (PPAR*α*), PPAR gamma (PPAR*γ*), and PPAR beta/delta (PPAR*β*/*δ*) [13]. The activation of PPAR*α* mediates effects such as the catabolism of fatty acids through the stimulation of mitochondrial lipid oxidation [14]. In previous works [15, 16] we have shown that, *in vivo* activation of PPAR*α* modulates the cardiac production of ROS, as well as the expression of antioxidant enzymes. Fenofibrate, a PPAR*α* agonist, has been clinically used for more than 30 years to reduce triglycerides and cholesterol levels in patients at risk of cardiovascular disease and has shown successful protection against the deleterious effects of I/R in experimental settings [17, 18]. Remarkably, fenofibrate possesses PPAR*α*-mediated anti-inflammatory, antioxidant and antifibrotic effects that may account for its direct cardioprotective [10, 11]. In addition, fenofibrate supports cardiac function and improves postischemic functional recovery in diet-induced obese mice [19]. Alternatively, fenofibrate therapy prevents isoproterenol-induced myocardial infarction [20] but also, inhibits the reperfusion-induced cardiac arrhythmias in isolated rat hearts [21].

However, up to date, no study has analysed the effect of fenofibrate in cultured cardiomyocytes undergoing H/R, HG and the combination of these conditions (HG+H/R). This latter could allow us to reproduce *in vivo* observed features and could be an *in vitro* model for further studies. Therefore, the aim of the study is to characterize the effects of fenofibrate treatment on oxidative stress and structural damage in cardiomyocytes subjected to hypoxia-reperfusion, high glucose and the combination of both conditions.

## 2. Materials and Methods

### 2.1. Animals

Newborn male and female Wistar rats (1-3 days), provided by the animal facilities of the Center for Research and Advanced Studies of the National Polytechnic Institute (CINVESTAV), were used to obtain cardiomyocytes. The protocol was carried out following the guidelines of the Institutional Ethics Committee (protocol number 0270/18), as well as those of the Official Mexican Standard for the use and care of laboratory animals NOM-062-ZOO 1999.

### 2.2. Neonatal Rat Cardiomyocyte (NRCM) Isolation and Culture

**Figure 1:**
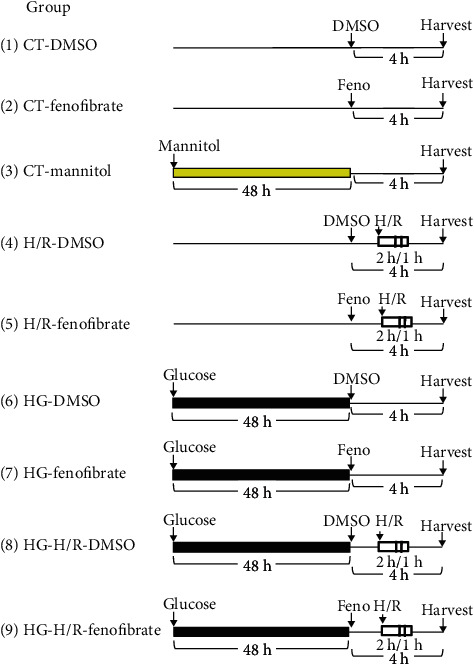
Schematic representation of the experimental group design. Primary cardiomyocyte cultures were divided into 9 groups. Mannitol (19.5 mM) and high glucose (25 mM) were administered 48 hrs before fenofibrate (10 *μ*M) or DMSO (0.1%) treatment. Exposition time for either fenofibrate or DMSO treatment was 4 hrs, with or without hypoxia-reperfusion maneuver, before harvest. CT = control; H/R = hypoxia-reperfusion; HG = high glucose; DMSO = dimethylsulfoxide 0.1%; Feno = fenofibrate 10 *μ*M. Yellow bar represents mannitol-treatment period, black bar represents glucose-treatment period, white bar represents hypoxia period, and dashed bar represents reoxygenation period.

As previously described [22], NRCMs were isolated from the heart of 1- to 3-day-old Wistar rats. Extirpated hearts were minced, and ventricles were digested four times for fifteen minutes each in trypsin (0.25% Invitrogen, Carlsbad, CA, USA) in a sterile environment. Cells were incubated for 90 minutes in cell culture flasks to allow noncardiac myocytes (mainly cardiac fibroblasts) to adhere to the plastic. NRCMs were cultured in F-10 medium (1x) nutrient mixture (HAM) with L-glutamine (Gibco, Waltham, MA, USA) containing 5.5 mmol/L of D-glucose supplemented with 10% heat-inactivated fetal bovine serum (FBS, Invitrogen, Carlsbad, CA, USA), 100 U/mL of penicillin, and 100 mg/L of streptomycin (Gibco, Waltham, MA, USA). NRCMs (1 × 10^6^ cells) were placed in a six-well culture plate and incubated at 37°C in a humidified atmosphere (5% CO_2_/95% O_2_). Experiments were performed on beating and confluent monolayers on the 3rd to 5th day of culture. Cell cultures were subdivided into the following experimental groups: (1) control treated with vehicle DMSO (0.1%) (control-DMSO), (2) control treated with fenofibrate (10 *μ*M) (control-Feno), (3) control treated with mannitol (19.5 mM) (control-mannitol), (4) hypoxia/reperfusion treated with DMSO (0.1%) (H/R-DMSO), (5) H/R treated with fenofibrate (10 *μ*M) (H/R-Feno), (6) high glucose (25 mM) treated with DMSO (0.1%) (HG-DMSO), (7) high glucose (25 mM) treated with fenofibrate (10 *μ*M) (HG-Feno), (8) high glucose (25 mM) plus hypoxia/reperfusion treated with DMSO (0.1%) (HG+H/R-DMSO), and (9) high glucose 25 mM plus hypoxia/reperfusion treated with fenofibrate (10 *μ*M) (HG+H/R-Feno) (Figure 1). High glucose was produced by incubating cells for 48 hours in F-10 medium containing 25 mmol/L of glucose ((+)D-glucose at 200 g/L, Gibco, Waltham, MA, USA). To produce H/R, cell cultures were covered with a coverslip for two hours [23, 24]. After that, the coverslip was removed and the cells were reoxygenated for 1 hour. Vehicle (DMSO, 0.1%) or fenofibrate (10 *μ*M, purity ≥ 99%, Sigma-Aldrich, St. Louis, MO, USA) were administered according to Figure 1.

In order to explore hyperosmolarity, cells were exposed to mannitol (19.5 mM, D-mannitol, Sigma-Aldrich, St. Louis, MO, USA).

### 2.3. Cell Viability

Cell viability was carried out according to Strober and Crowley et al. [25, 26]. A volume of 0.1 mL of trypan blue 0.4% was added to 1 mL of cells (1 × 10^6^ cells). An aliquot of 50 *μ*L of cells was loaded on a Neubauer chamber (Neubauer, Marienfeld, 0.0025 mm^2^; Wöllerspfad Lauda-Königshofen, Germany) and immediately examined under a microscope at 10x magnification. The amount of blue-stained cells (dead) and the total number of cells were counted. The cell viability must be at least 95% to consider a healthy logarithmic phase culture.

### 2.4. Real-Time PCR

Using the TRIzol Reagent (Thermo Fisher Scientific, Rockford, IL, USA), RNA was isolated from the equivalent of 5 × 10^6^ cells from the different experimental groups. The integrity of the RNA was checked on a 1% agarose gel. First, a reaction with reverse transcriptase (RT) with 2.5 *μ*g of total RNA in the presence of 200 U of reverse transcriptase (RT) from the Moloney Murine Leukemia Virus (MMLV) (Invitrogen, USA) was carried on. The amount of HIF-1*α* mRNA was quantified by real-time PCR in the ABI Prism 7000 Sequence Detection System (TaqMan, ABI, Foster City, CA, USA) through the reaction with SYBR Green. As an endogenous control, GAPDH was used and the sequences of the HIF-1*α* harvesters used were the following: ATACCAGCAGTAACCAGCCG (sense) and CTGTGGCTGAGAGTCCTTCG (antisense). The method to calculate the amount of HIF-1*α* was ΔCT (increase of the cycle threshold) [27].

### 2.5. ROS Production

Cardiomyocytes (1 × 10^6^) from the different experimental groups were washed with PBS and incubated for 30 min in the darkness with the CellRox™ Green Reagent (Thermo Fisher Scientific, Waltham, MA, USA) at a final concentration of 5 *μ*M. After incubation, the medium was removed, the cells were washed twice with PBS, scraped off with 1 mL of PBS, and placed in dark Eppendorf tubes. Subsequently, the fluorescence emitted by the interaction of the free radicals with the CellRox indicator was determined by a flow cytometer (FACSCalibur model, BD Biosciences, San Jose, CA, USA) and the CellQuest analysis program (BD Biosciences, San Diego, CA, USA). The results were calculated as the geometric mean fluorescence (MF) in FL1 of 3000 events, obtained by region and its fluorescence histograms, where the displacement of the fluorescence peaks is observed depending on the treatment in each cell group loaded with the CellRox indicator, all of them compared with the intrinsic fluorescence of a group of cells that were incubated without the indicator [28, 29].

### 2.6. Protein Expression by Western blot

Total protein content in the cultures was quantified by the BCA Protein Assay Kit (Pierce, Waltham, MA, USA) as previously reported [30]. Protein from cell lysates (80 *μ*g of protein) were subjected to separation on a 12% SDS-PAGE gel (100 V) for 2 hours, followed by electrotransfer to a polyvinylidene fluoride (PVDF) membrane (0.45 *μ*m, Millipore, Billerica, MA, USA) at 10 V for 1 hour. In order to block unspecific binding, membranes were incubated with 5% nonfat milk (Bio-Rad, Hercules, CA, USA) in PBS-Tween 0.1% as reported elsewhere. Blots were probed with specific antibodies against *β*-actin (1 : 5000), HIF-1*α* (1 : 100), SOD Cu^2+^/Zn^2+^ (1 : 100), SOD Mn^2+^ (1 : 100), p47phox (1 : 100), NOX4 (1 : 100), eNOS (1 : 100), p-eNOS^Ser 1177^ (1 : 100), PPAR*α* (1 : 50), Nrf2 (1 : 50), Keap1 (1 : 50) (Santa Cruz Biotechnology Inc., Santa Cruz, CA, USA), and p-PPAR*α*^Ser12^ (Abcam, Cambridge, UK). Signals were detected by the Immobilon Western Chemiluminescent HRP Substrate (Millipore, Billerica, MA, USA). Images from each film were acquired by a GS-800 densitometer (including Quantity One software from Bio-Rad Laboratories, Inc., Hercules, CA, USA). Blots were stripped and reincubated with *β*-actin antibody as load control. Values of each band density are expressed as arbitrary units.

### 2.7. Antioxidant Capacity Assay

Total antioxidant capacity was determined by the method described by Apak et al. [31]. Briefly, a suspension of 6 × 10^6^ cells previously centrifuged 1500 rpm/10 min was diluted with 145 *μ*L of 0.1 M phosphate buffer at pH 7.5 and shook at 500 rpm for 200 sec. 100 *μ*L of the diluted sample was further treated with 50 *μ*L of 0.01 M CuCl_2_ and shook at 500 rpm for 200 sec. Then, 50 *μ*L of 0.01 M batocuproin was added and vortexed again at 500 rpm for 200 sec. The concentration of Cu^2+^ reduced to Cu^+^ was measured by means of a spectrometer to 490 nm (DW2000, SLM-Aminco, Urbana, IL, USA). Total antioxidant capacity is expressed as *μ*mol/L of Cu^2+^ reduced to Cu^+^ and is calculated as follows:

(1) $$\begin{array}{c} \text{TAC}=\left(\Delta F_{\text{em}}\right)\left(\text{DF}\right)\;\;\left(6.418629\;\;\mu \text{mol}/\text{L}\right), \end{array}$$

where TAC is the total antioxidant capacity,∆*F*_em_ is the flourescence difference emitted (treated sample − diluted sample), DF is the dilution factor (DF = 8), and the kinetics factor of extinction-emission for the Cu^2+^ batocuproin complex is 641.8629 *μ*mol/L.

### 2.8. Quantification of Malondialdehyde

Malondialdehyde (MDA) was determined by capillary zone electrophoresis in cardiomyocytes (6 × 10^6^ cells) obtained from every experimental group, as described by Sánchez-Aguilar et al. [30]. The sample was deproteinized with cold methanol (1 : 1), centrifuged at 16,000 × *g* for 15 minutes, and filtered through a 0.22 *μ*m nitrocellulose membrane filter (Millipore, Billerica, MA, USA). It was diluted (1 : 10) with cold sodium hydroxide (0.1 M) and analyzed in a P/ACE™ MDQ Capillary Electrophoresis System (Beckman Coulter, California, USA) under the following conditions: The samples were injected under hydrodynamic pressure at 0.5 psi/10 s. The separation was performed at -25 kV for 4 min at 267 nm. The capillary was washed between runs with 0.1 M NaOH for 2 minutes, distilled water for 2 min, and phosphate buffer for 4 minutes. The concentration of MDA was expressed in *μ*M and was determined interpolating with a standard curve.

### 2.9. Quantification of 8-Hydroxy-2′-Deoxyguanosine

8-Hydroxy-2′-deoxyguanosine (8-OH-2dG) was determined by capillary zone electrophoresis and UV detection, by diode array as described by Sánchez-Aguilar et al. [30]. A sample of cardiomyocytes (6 × 10^6^ cells) from each experimental group was deproteinized with 20% trichloroacetic acid (10 : 1). It was centrifuged at 16,000 × *g* for 15 minutes and filtered through 0.22 *μ*m nitrocellulose membrane filters. Samples were analyzed with the P/ACE™ MDQ Capillary Electrophoresis System (Beckman Coulter, CA, USA). The capillary was preconditioned passing a solution of 2 M sodium hydroxide for 30 min, then deionized water for 30 min, and finally running buffer (10 mM borates, pH 9.0) for 30 min. The sample was injected under hydrodynamic pressure at 0.5 psi/10 s. The separation was carried out at 20 kV for 8 min at 200 nm. The capillary was washed between runs with 2 M NaOH for 2 min and distilled water for 2 min. The results were expressed in pmol/mL. The concentration of 8-OH-2dG was determined interpolating the values with a standard curve.

### 2.10. Capillary Zone Electrophoresis for Determination of BH_4_ and BH_2_

The contents of BH_4_ and BH_2_ in cardiomyocytes from every experimental group were determined as described by Ibarra-Lara et al. [16]. Briefly, 50 *μ*L of sample containing 6 × 10^6^ cells was deproteinized with cold methanol (1 : 1 *v*/*v*) and centrifuged at 16,000 × *g* for 15 min at 10°C and filtered with a nitrocellulose membrane (0.22 *μ*m, Millipore, Billerica, MA, USA). Measurement was performed using a P/ACE™ MDQ Capillary Electrophoresis System (Beckman Coulter, Mexico City, Mexico), with laser-induced fluorescence detection. A standard curve of BH_2_ and BH_4_ (Sigma-Aldrich, St. Louis, MO, USA) was used to calculate concentrations. Data are expressed as pmol/mg of protein of BH_4_ and BH_2_.

### 2.11. Electron Microscopy

Electron microscopy was used to evaluate the ultrastructure of cardiomyocytes. We followed the method described by Ibarra-Lara et al. [16] using 1 × 10^6^ cells. The evaluation was carried out with a JEM 1011 (JEOL Ltd., Tokyo, Japan) at 60 kV. The magnification employed was 50,000x.

### 2.12. Data Analysis

Data obtained is presented as the mean ± standard error of the mean of 6 independent experiments. Differences between the groups were analyzed by two-way analysis of variance (two-way ANOVA) followed by Tukey's post hoc test. Statistical significance was accepted when *p* < 0.05. For comparisons between two groups, an unpaired Student's *t*-test was used.

## 3. Results

### 3.1. HIF-1*α* Evaluation

**Figure 2:**
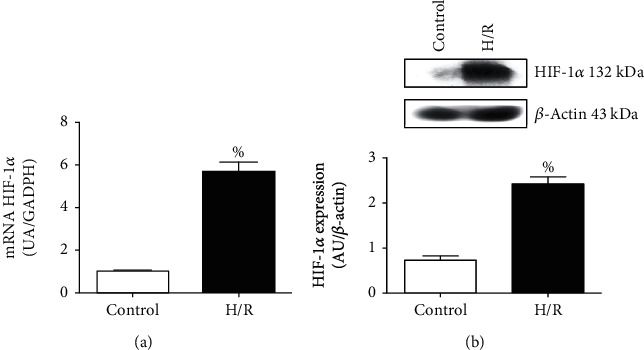
Evaluation of the efficiency of the coverslip assay by quantification of HIF-1*α* in primary cultures of cardiomyocytes undergoing hypoxia/reperfusion (H/R). (a) Quantification of mRNA HIF-1*α* performed through qPCR. HIF-1*α* sense: ATACCAGCAGTAACCAGCCG; antisense: CTGTGGCTGAGAGTCCTTCG. (b) Protein expression of HIF-1*α* by Western blot and quantified by densitometric analysis. The values represent the mean ± standard error of the mean (SEM) of 5 different experiments. ^%^*p* < 0.05 vs. control. Student's unpaired*t*-test.

To determine if the method used to induce hypoxia (coverslip) was effective, we determined the expression of HIF-1*α*. This determination was carried out through qPCR and Western blot. Our results show that both mRNA and protein HIF-1*α* were significantly increased in cultures undergoing hypoxia-reperfusion compared to control (Figures 2(a) and 2(b)). These results suggest that the method employed to generate hypoxia (coverslip) was effective.

### 3.2. Evaluation of Cell Viability

**Figure 3:**
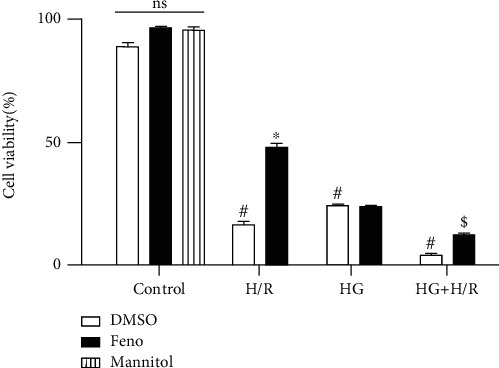
Effect of fenofibrate (10 *μ*M) on cell viability. Cell viability was assessed by a trypan blue dye exclusion test of control, hypoxia/reperfusion (H/R), high glucose (HG), or the combination of conditions (HG+H/R) in primary culture of cardiomyocytes. DMSO = dimethylsulfoxide (0.1%); Feno = fenofibrate (10 *μ*M). ^#^*p* < 0.05 vs. control-DMSO; ^∗^*p* < 0.05 vs. H/R-DMSO; ^$^*p* < 0.05 vs. HG+H/R-DMSO. Values represent the mean ± SEM of 6 different experiments. Two-way analysis of variance (ANOVA) followed by Tukey's post hoc test.

To determine cell viability, we used the trypan blue dye technique. Accordingly, our results indicate that cardiomyocytes under DMSO, fenofibrate, or mannitol exhibited cell viability above 95%, indicating that cell cultures were in optimal conditions and that treatments exerted no harm. On the other hand, cardiomyocytes exposed to H/R, HG, and HG+H/R exhibited lower cell viability compared to the control groups. Fenofibrate administration in the H/R group was able to prevent cell death. In addition, we observed that HG cells exposed to fenofibrate exhibited a viability comparable to those HG-DMSO-exposed cardiomyocytes (Figure 3), a result that suggests a lack of fenofibrate-induced effect once the hyperglycemia-induced damage is done. It is worth mentioning that mannitol did not induce loss of cell viability, ruling out hyperosmolarity as the responsible process for cell death and confirming the harmful effect of high glucose *per se*. The fenofibrate effect can be observed in the group with both conditions where it increased cell viability; this result was different from the HG+H/R-DMSO group, suggesting that fenofibrate could be helpful to preserve cell viability after H/R although high glucose is present.

### 3.3. Production of ROS

**Figure 4:**
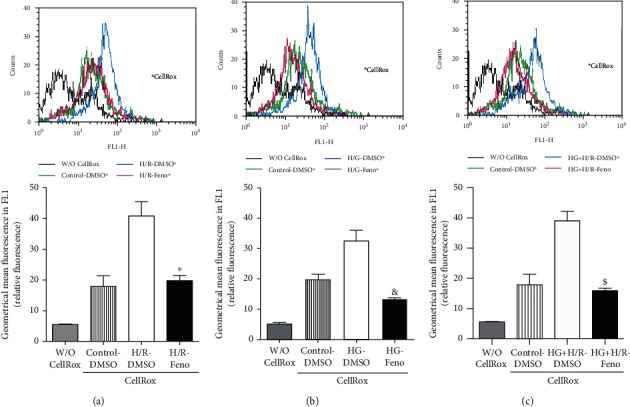
Electropherograms and quantification of ROS production by flow cytometry in primary cultures of cardiomyocytes under (a) hypoxia/reperfusion (H/R), (b) high glucose (HG), or (c) HG+H/R. ^∗^*p* < 0.05 vs. H/R-DMSO; ^&^*p* < 0.05 vs. HG-DMSO; ^$^*p* < 0.05 vs. HG+H/R-DMSO. W/O CellRox = blank; CellRox = vehicle; DMSO = dimethylsulfoxide (0.1%); Feno = fenofibrate (10 *μ*M). Values represent the mean ± SEM of 6 different experiments. Two-way analysis of variance (ANOVA) followed by Tukey's post hoc test.

As shown in Figure 4, the effect of fenofibrate on ROS production was determined in H/R, HG, and HG+H/R conditions. Our results show that the H/R, HG, and the coexistence of both conditions significantly increased ROS production. Fenofibrate treatment decreased ROS production despite the presence of pathological conditions (Figures 4(a)–4(c)).

### 3.4. Evaluation of the Antioxidant Effect

**Figure 5:**
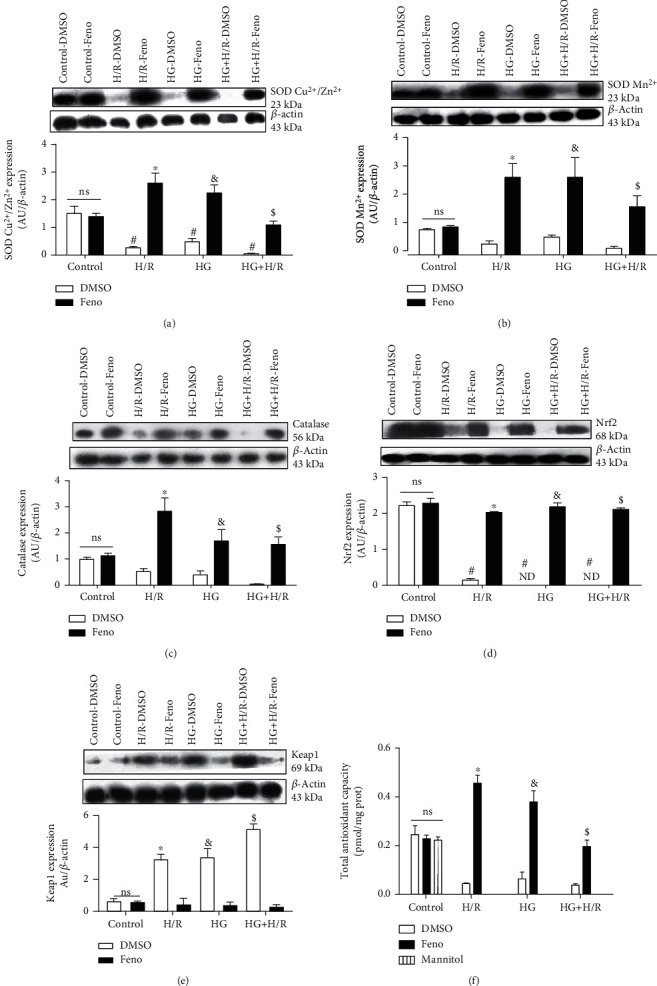
Fenofibrate (10 *μ*M) increased antioxidant defense in primary cultures of cardiomyocytes subjected to hypoxia/reperfusion (H/R), high glucose (HG), or the combination of conditions (HG+H/R). Protein expression by Western blot and densitometric analysis of (a) SOD Cu^2+^/Zn^2+^, (b) SOD Mn^2+^, (c) catalase, (d) Nrf2, (e) Keap1, and (f) total antioxidant capacity. ND = not detectable; DMSO = dimethylsulfoxide (0.1%); Feno = fenofibrate (10 *μ*M). ^#^*p* < 0.05 vs. control-DMSO; ^∗^*p* < 0.05 vs. H/R-DMSO; ^&^*p* < 0.05 vs. HG-DMSO; ^$^*p* < 0.05 vs. HG+H/R-DMSO. Values represent the mean ± SEM of 6 different experiments. Two-way analysis of variance (ANOVA) followed by Tukey's post hoc test.

To explore the role of PPAR*α* in antioxidant defense, the expression of SOD Cu^2+^/Zn^2+^, SOD Mn^2+^, and catalase was measured in the different groups. The results obtained show that the expression of antioxidant enzymes decreased in the HG, H/R, and HG+H/R groups. Interestingly, treatment with fenofibrate significantly increased the expression of SODs and catalase (Figures 5(a)–5(c)). These results suggest that the activation of PPAR*α* by fenofibrate decreased oxidative stress due to the increased expression of antioxidant enzymes. In order to investigate further the antioxidant mechanism of fenofibrate, the expression of the Keap1/Nrf2 pathway was determined. Our results indicate that cardiomyocytes, under pathological conditions (HG, H/R, and HG+H/R), exhibited a lower expression of Nrf2, while fenofibrate increased its expression (Figure 5(d)). Fenofibrate treatment decreased Keap1 expression (Figure 5(e)). As expected, treatment with fenofibrate increased the total antioxidant capacity in HG, H/R, and HG+H/R (Figure 5(f)). Therefore, fenofibrate favors an antioxidant environment in cultured cardiomyocytes.

### 3.5. Evaluation of the Expression of NADPH Subunits

**Figure 6:**
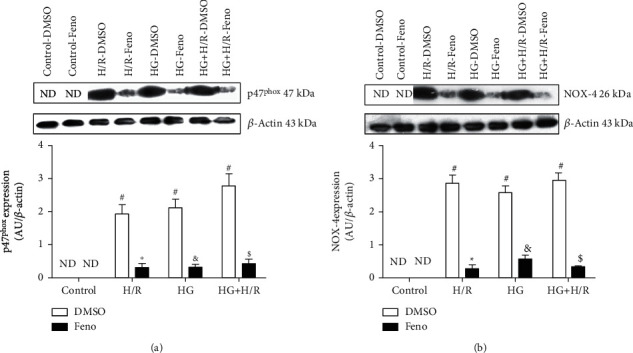
Fenofibrate (10 *μ*M) decreased the expression of the NADPH oxidase subunits in primary cultures of cardiomyocytes undergoing hypoxia/reperfusion (H/R), high glucose (HG), and the combination of both conditions (HG+H/R). (a) Protein expression and densitometric analysis of the p47phox subunit expression. (b) Protein expression and densitometric analysis of the NOX4 subunit expression. ND = not detectable; DMSO = dimethylsulfoxide (0.1%); Feno = fenofibrate (10 *μ*M). ^#^*p* < 0.05 vs. control-DMSO; ^∗^*p* < 0.05 vs. H/R-DMSO; ^&^*p* < 0.05 vs. HG-DMSO; ^$^*p* < 0.05 vs. HG+H/R-DMSO. Values represent the mean ± SEM of 6 different experiments. Two-way analysis of variance (ANOVA) followed by Tukey's post hoc test.

Due to the relevance of NADPH oxidase p47phox- and NOX4-subunits, we evaluated their expression. In control cardiomyocytes, we did not observe the expression of these proteins. Interestingly, in H/R, HG, and HG+H/R-exposed cardiomyocytes, the expression of both NADPH subunits significantly increased. Remarkably, this effect was prevented by fenofibrate (Figures 6(a) and 6(b)).

### 3.6. Effect of Fenofibrate on Malondialdehyde, 8-Hydroxy-2-Desoxyguanosine, BH_4_, and BH_2_

**Figure 7:**
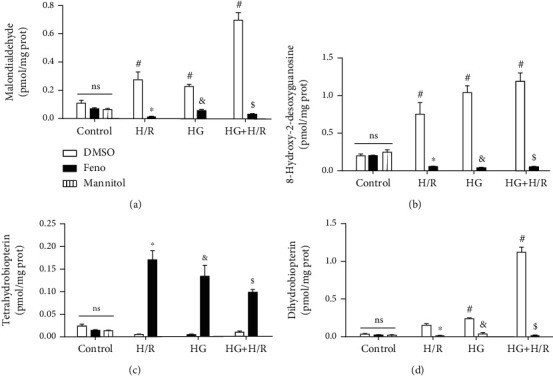
Fenofibrate (10 *μ*M) decreased the production of oxidant markers and prevented BH_4_ oxidation in primary cultures of cardiomyocytes undergoing hypoxia/reperfusion (H/R), high glucose (HG), and HG+H/R. (a) Malondialdehyde, (b) 8-hydroxy-2-deoxyguanosine, (c) BH_4_, and (d) BH_2_ levels were measured by capillary zone electrophoresis. DMSO = dimethylsulfoxide (0.1%); Feno = fenofibrate (10 *μ*M). ^#^*p* < 0.05 vs. control-DMSO; ^∗^*p* < 0.05 vs. H/R-DMSO; ^&^*p* < 0.05 vs. HG-DMSO; ^$^*p* < 0.05 vs. HG+H/R-DMSO. Values represent the mean ± SEM of 6 different experiments. Two-way analysis of variance (ANOVA) followed by Tukey's post hoc test.

Reactive oxygen species may interact with several targets in the cell. Likewise, lipids, DNA, and metabolites are oxidized by their action, modifying their physiological role. The lipid damage indicator, MDA, was found increased in the H/R, HG, and HG+H/R groups, compared to control. In addition, MDA decreased significantly in every cardiomyocyte culture (H/R, HG, and HG+H/R) incubated with fenofibrate (Figure 7(a)). The effect on DNA was also evaluated through the presence of 8-hydroxy-2-desoxyguanosine (8-OH-2dG), an essential marker of oxidative damage on DNA. Our results indicate that this parameter increased in the H/R, HG, and HG+H/R groups. Interestingly, the stimulation of PPAR*α* by fenofibrate significantly decreased the values of this DNA damage marker (Figure 7(b)). In order to produce nitric oxide (NO), endothelial nitric oxide synthase (eNOS) requires cofactors such as BH_4_. However, this molecule is highly susceptible to be oxidized to BH_2_, promoting an uncoupled state in eNOS being unable to synthesize NO and instead producing O_2_^•-^. Therefore, the concentrations of BH_4_ and BH_2_ were determined by capillary zone electrophoresis. The results show that either H/R, HG, or the coexistence of both experimental conditions induced a significant decrement of BH_4_ levels. In addition, treatment with fenofibrate prevented the oxidation of BH_4_ (Figure 7(c)). On the other hand, BH_2_ levels increased in the HG and HG+H/R groups. Again, fenofibrate maintained this parameter at levels similar to the control group (Figure 7(d)). These results suggest that fenofibrate favors an eNOS-coupled state due to the lower oxidation of BH_4_ in a process in which oxidative stress is involved.

### 3.7. Evaluation of the Expression of eNOS and P-eNOS^Ser1177^

**Figure 8:**
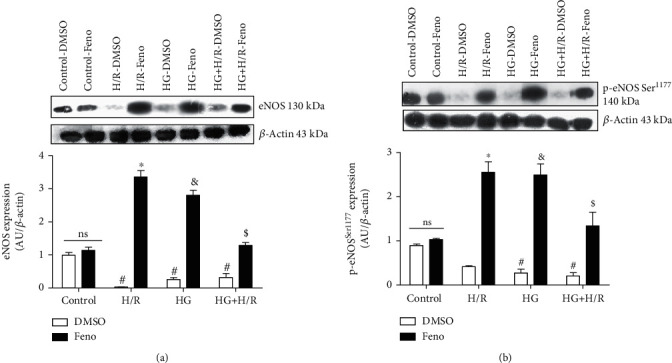
Fenofibrate (10 *μ*M) increases the expression of endothelial nitric oxide synthase (eNOS) and p-eNOS^Ser1177^ in primary cultures of cardiomyocytes undergoing hypoxia/reperfusion (H/R), high glucose (HG), and HG+H/R. Protein expression and densitometric analysis of (a) total eNOS and (b) phospho-eNOS^Ser1177^ were performed. DMSO = dimethylsulfoxide (0.1%); Feno = fenofibrate (10 *μ*M). ^#^*p* < 0.05 vs. control-DMSO; ^∗^*p* < 0.05 vs. H/R-DMSO; ^&^*p* < 0.05 vs. HG-DMSO; ^$^*p* < 0.05 vs. HG+H/R-DMSO. Values represent the mean ± SEM of 6 different experiments. Two-way analysis of variance (ANOVA) followed by Tukey's post hoc test.

Figure 8 shows the effect of fenofibrate on eNOS and p-eNOS^Ser1177^expression in the HG, H/R, and HG+H/R groups. Our results show that eNOS expression is affected by HG, H/R, and the combination of pathological factors at both nonphosphorylated (eNOS) and phosphorylated (p-eNOS^Ser1177^) forms exhibited as a reduction in their expression. As expected, fenofibrate treatment significantly increased the expression of eNOS and p-eNOS ^Ser1177^ suggesting that the PPAR*α* activator potentially increases NO-bioavailability (Figures 8(a) and 8(b)).

### 3.8. Evaluation of the Expression of PPAR*α* and p-PPAR*α*^Ser12^

**Figure 9:**
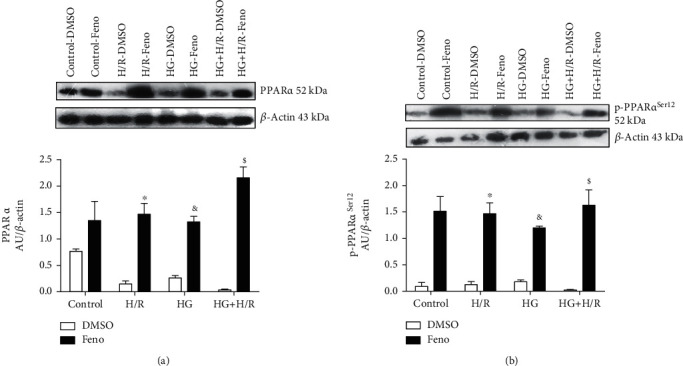
Fenofibrate (10 *μ*M) increases peroxisome proliferator-activated receptor alpha (PPAR*α*) and phospho-PPAR*α*^Ser12^ expression in primary cultures of cardiomyocytes undergoing hypoxia/reperfusion (H/R), high glucose (HG), and HG+H/R. Protein expression and densitometric analyses of (a) PPAR*α* and (b) phospho-PPAR*α*^Ser12^ were performed. DMSO = dimethylsulfoxide (0.1%); Feno = fenofibrate (10 *μ*M). ^#^*p* < 0.05 vs. control-DMSO; ^∗^*p* < 0.05 vs. H/R-DMSO; ^&^*p* < 0.05 vs. HG-DMSO; ^$^*p* < 0.05 vs. HG+H/R-DMSO. Values represent the mean ± SEM of 6 different experiments. Two-way analysis of variance (ANOVA) followed by Tukey's post hoc test.

In order to probe that fenofibrate was able to stimulate PPAR*α*, we evaluated its expression as well as p-PPAR*α*^Ser12^, a marker of fibrate action. As shown on Figure 9(a), the H/R, HG, and HG+H/R groups have a downward trend of PPAR*α* expression. In Figure 9(b), we can observe a low profile of p-PPAR*α*Ser12 expression in the H/R, HG, and HG+H/R groups. However, fenofibrate significantly increased the expression of PPAR*α* and p-PPAR*α*^Ser12^, suggesting that fenofibrate was able to reach its target and activate it.

### 3.9. Evaluation of the Ultrastructure of Cardiomyocytes

**Figure 10:**
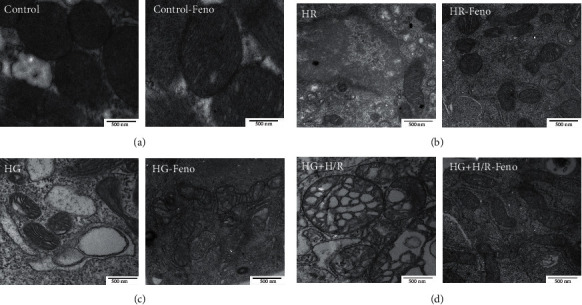
Fenofibrate (10 *μ*M) attenuates damage to the mitochondrial ultrastructure in primary cultures of cardiomyocytes subjected to hypoxia/reperfusion (H/R), high glucose (HG), and HG+H/R. Details of the ultrastructure by electron microscopy: 500 nm, 50,000x. (a) Control-DMSO and control/fenofibrate, (b) H/R-DMSO and H/R-fenofibrate, (c) HG-DMSO and HG-fenofibrate, and (d) HG+H/R-DMSO and HG+H/R-fenofibrate. The images are representative of 6 experiments per group.

It was observed that in the control and control-fenofibrate groups, there was an homogeneous distribution of mitochondria, as well as a continuous membrane (Figure 10(a)). Cardiomyocytes subjected to H/R exhibited tubular mitochondrial ridges and were less dense than controls. In addition, there was an emptying of its content and the membranes exhibited discontinuous borders. On the other hand, fenofibrate improved the condition of mitochondria in cultured cells exposed to H/R, being observed dense although slightly reduced in size and elongated. In this case, the inner and outer membranes exhibited continuous borders. These characteristics indicate that mitochondria were functional (Figure 10(b)). Exposure to HG caused smaller and swollen mitochondria with signs of vacuolation (Figure 10(c)). In the HG-fenofibrate-exposed cardiomyocytes, it was observed that mitochondria were normal in size and density, indicating that the mitochondria were functional. Cardiomyocytes subjected to HG+H/R exhibited small and lysed mitochondria, further showing that the coexistence of both experimental conditions damages the ultrastructure of cardiomyocytes. Cardiomyocytes exposed to HG+H/R and treated with fenofibrate were small but exhibited dense mitochondria, without disruption of their membrane. These results indicate that fenofibrate protected the cardiomyocyte, especially mitochondria from HG and H/R-induced damage (Figure 10(d)).

## 4. Discussion

In the present work, we established an *in vitro* model using neonatal rat cardiomyocytes exposed to HG, H/R, and the combination of both conditions to mimic the pathological features present in diabetes mellitus and myocardial infarction. Under these conditions, we demonstrated that treatment with fenofibrate exerts a protective effect in cardiomyocytes by promoting an antioxidant environment and preserving the mitochondrial ultrastructure, through the upregulation of the master antioxidant genes *PPARα* and *Nrf2*, events that contribute to the attenuation of cell death caused by HG+H/R injury.

Several experimental models reproduce, at different levels, the pathological conditions present in diabetes mellitus (DM) and myocardial infarction (MI). When performing cell cultures, the physiological, biochemical, and genetic properties of the cells are maintained to the maximum. In addition, the characteristic architecture of the tissue is maintained, preserving cellular interactions. The advantage of cell culture over other models lies in the precise and fine control of experimental conditions in the cellular environment (pH, temperature, osmotic pressure, oxygen levels, CO_2_, etc.) [32]. As reported by Pitts and Toombs, hypoxia/reperfusion in cell cultures can be produced by placing a coverslip on top of the media; it creates a barrier to the diffusion of oxygen, immediately producing hypoxia and metabolic by-products, thus resembling the conditions of ischemia *in vivo*. Meanwhile, reperfusion is achieved by restoring oxygen concentrations [23].

Hyperglycemia is an important risk factor for acute myocardial infarction. It enhances oxidative stress, stimulates nitric oxide synthase uncoupling, increases mitochondrial derangement, and impairs prosurvival cell signaling in the diabetic myocardium, making it more vulnerable to ischemia-reperfusion injury [33]. Luan et al. identified that HG treatment sensitized adult cardiomyocytes to ischemia/reperfusion (I/R) injury [34].

Myocardial hypoxia results in metabolic changes and irreversible damage leading to cardiomyocyte death [35]. Hypoxia not only damages the heart but other organs as well; fenofibrate has been tried and used as the drug of choice to reduce the effects of H/R in various organs, as demonstrated by Bhalodia et al., who provided evidence that fenofibrate exerted renoprotective effects on hypoxia/reperfusion injury (H/R). Therefore, this drug not only protects the heart from H/R but also protects the kidney subjected to the same conditions [36]. Fenofibrate has been reported to promote an antioxidant, anti-inflammatory, and anti-ischemic effect in a model of intestinal IR injury in rats [37], improving the intestinal recovery and the enterocyte turnover. Furthermore, studies have highlighted the pleiotropic vascular endothelial protective and antihypertensive actions of fenofibrate [38].

In our study, the success of the technique used to produce hypoxia was verified evaluating HIF-1*α* expression. HIF-1*α* is a transcription factor that regulates the cellular response to hypoxia and acts as a regulator of oxygen homeostasis. Our results indicate that the method used to produce hypoxia (coverslips) significantly increased the expression of this factor in cell cultures subjected to hypoxia (Figures 2(a) and 2(b)). This result is in agreement with that reported by Jia et al. [39]. They studied a rat model of intestinal ischemia/reperfusion (I: 1 hour/R: 2 hours), and their results show that the expression of HIF-1*α* increased in rats with I/R compared to controls.

It is widely reported that H/R causes cell death. A well accepted technique to evaluate this event is the cellular exclusion of trypan blue dye used by Zhang et al. [6], where H9c2 cells were subject to H/R and HG (55 mmol/L). Our results reproduce the detrimental effects of HG and H/R, reported by Zhang et al., and exhibit the extension of the event when the two pathological factors coexist. We observed that in those cardiomyocytes exposed 48 hours to HG and lately to fenofibrate (4 hours), there was no protection (Figure 3). Probably, the lack of effect exerted by fenofibrate would be related to hyperglycemia-induced metabolic memory. This metabolic memory increases fibronectin, inflammatory mediators, vascular endothelial growth factor, and intercellular adhesion molecule-1 (ICAM-1); these effects are sustained following normalization of glucose levels in the window of days to weeks [40, 41]. According to Kim et al. [42], chronic high glucose reduced PPAR binding to target genes.

Our results showed that H/R, HG, and the combination of both conditions increased the production of ROS (Figure 4). This result is similar to that obtained by Wang et al. [43] who observed higher levels of ROS in H9C2 cells subjected to H/R compared to those of control cell cultures. The authors also reported the diminished expression of SOD Mn^2+^, resulting in oxidative damage to the cardiomyocytes. A similar result was obtained by Zhou et al. [44] in cardiomyocytes from Sprague-Dawley rats exposed to HG (30 mmol/L). Our research extended those observations to the combination of both pathological conditions and the effect of fenofibrate treatment on oxidative stress, showing increased antioxidant enzyme expression as well as raised antioxidant capacity (Figures 5(a), 5(b), 5(c), and 5(f)). The expression of Keap1/Nrf2 was also evaluated. The transcription factor Nrf2 regulates the inducible expression of numerous detoxifying and antioxidant genes. It binds to a specific DNA sequence known as ARE (Antioxidant Response Element) that can be activated by several electrophils and oxidant compounds of diverse chemical nature. Nrf2 activation is constitutively repressed by its binding with a cytosolic protein known as Keap1 and to the cytoskeleton. This interaction promotes the permanent Nrf2 degradation by the proteosome, implying that the primary control of Nrf2 function lies on its subcellular distribution rather on its de novo synthesis. Additionally, it has been suggested that the Keap1/Nrf2 system contributes to the protection in pathologies like DM and ischemia. In fact, the Nrf2-mediated antioxidant response is one of the main cellular defense mechanisms that facilitates cell survival under toxic attacks [45]. Our results indicate that this factor decreases in HG, H/R, and the coexistence of both conditions; however, treatment with fenofibrate significantly increased its expression (Figure 5(d)). He et al. [45] cultured cardiomyocytes from Nrf2^−/−^ knockout (KO) and wild-type (WT) mice and exposed them to HG levels (40 mM). Cardiomyocytes lacking the expression of Nrf2 exhibited a remarkably high level of ROS compared to WT cardiomyocyte cultures. Nrf2 also plays a role in cardiac protection against H/R. Xu et al. [46] showed that Nrf2 KO mice developed a larger infarct size posterior to ischemia/reperfusion. In addition, a recent study [47] showed that fenofibrate attenuates oxidative stress in diabetic retinopathy through Keap1/Nrf2, suggesting that cardiomyocytes stimulated by fenofibrate were protected from oxidative stress following a pathway that includes Nrf2. Our results also showed that Keap1 is increased in cardiomyocytes with pathological conditions, while treatment with fenofibrate decreased its expression. This result is similar to that found by Wu et al. [48], where PPAR*α* regulates the pathway Keap1/NRF2.

One of the most active enzymes in ROS production is NADPH oxidase [49, 50]. Our results show that cardiomyocytes exposed to H/R, HG, and HG+H/R overexpressed NADPH oxidase subunits, namely, 47phox and NOX4, an event that was decreased by fenofibrate stimulation (Figure 6). Current results agree with previous reports from our laboratory showing that clofibrate treatment to Wistar rats subjected to myocardial infarction reduces the expression of NADPH oxidase subunits [15].

In our study, we assessed the damage to important macromolecules in cells such as lipids and DNA. Our results indicate that H/R, HG, and HG+H/R-induced damage to lipids and DNA increased (Figures 7(a) and 7(b)), as well as the oxidation of the cofactor BH_4_, which is observed as an increased content of BH_2_ (Figures 7(c) and 7(d)). The stimulation with fenofibrate reduced the oxidative damage to those molecules. Previous studies [51, 52] show that the lower bioavailability of BH_4_ not only prevents the formation of NO by eNOS but also increases the formation of ROS, further producing damage to the cardiomyocytes. Additionally, previous reports [53] showed that hypoxia activates cellular proteases which may degrade eNOS. Goya et al. [54] demonstrated that WY14643, a PPAR*α* agonist, increased eNOS expression in bovine aortic endothelial cells. In agreement with the observations of Goya et al., our data show that cardiomyocytes exposed to fenofibrate raised eNOS and p-eNOS^Ser1177^despite being exposed to pathological conditions (HG, H/R, HG+H/R) (Figures 8(a) and 8(b)).

Importantly, it has been reported that the phosphorylation of PPAR*α* on Ser12 increases its activity and correlates with increased transactivation of PPAR*α* in cardiomyocytes [55]. Our results indicate that fenofibrate increases the expression of PPAR*α* and p-PPAR*α*^Ser12^, while H/R, HG, and the combination of both conditions decrease their expression (Figures 9(a) and 9(b)). The rise in p-PPAR*α*^Ser12^, and therefore the activation of PPAR*α*, correlates with the increased expression of the antioxidant enzymes (SOD and catalase) and transcription factors like Nrf2, which have been reported to contain a PPAR response element in their sequences. Consequently, the activation of PPAR*α* is fundamental for cardiac functions by promoting the homeostatic balance between ROS and antioxidants [56, 57].

Fenofibrate attenuated mitochondrial damage caused by HG, H/R, and the coexistence of both pathological factors (Figure 10). We hypothesize that the lower damage observed in cardiomyocytes is due to (1) decreased oxidative stress, a consequence of reduced expression of NADPH oxidase enzyme subunits and higher antioxidant defense, where Nrf2 plays an important role; (2) lower damage to macromolecules such as membrane lipids and DNA; and (3) higher expression of p-eNOS^Ser1177^. These events could contribute to mitochondrial protection, further supporting the reports of Holmstrom et al. [58], Li et al. [59], and Ilangovan et al. [60].

Currently, there are human studies proving the effectiveness of fenofibrate; for instance, the action to control cardiovascular risk in diabetes (ACCORD STUDY) demonstrated a 40% reduction in the progression of proliferative diabetic retinopathy in type 2 diabetic patients using fenofibrate treatment [61]. Fenofibrate intervention decreases the incidence of myocardial infarction and reduces the risk of subsequent clinical cardiovascular events in patients with type 2 diabetes [62].

However, the use of fenofibrate to reduce the incidence of cardiovascular events in diabetic patients has been controversial due to the heterogeneity of the treatment's response in clinical trials [63, 64].

**Figure 11:**
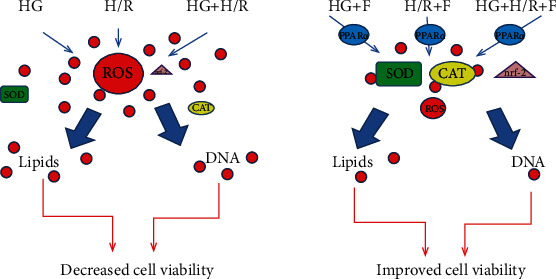
A schematic diagram proposing the mechanism of action of fenofibrate in H/R, HG, and HG+H/R in cardiomyocyte cell culture.

In summary, the effect of fenofibrate on the pathologies under study are illustrated in Figure 11.

## 5. Conclusions

According to our results, we can conclude that H/R, HG, and the coexistence of both pathologies generate oxidative stress in cardiomyocytes leading to lower cell viability. Further, fenofibrate treatment improves cell viability and favors an antioxidant environment contributing to preserve cardiac ultrastructure.

## Data Availability

The datasets generated during the study are available from corresponding author on reasonable request.
